# Efficacy and safety of chlorpromazine as an adjuvant therapy for glioblastoma in patients with unmethylated *MGMT* gene promoter: RACTAC, a phase II multicenter trial

**DOI:** 10.3389/fonc.2023.1320710

**Published:** 2023-12-14

**Authors:** Andrea Pace, Giuseppe Lombardi, Veronica Villani, Dario Benincasa, Claudia Abbruzzese, Ilaria Cestonaro, Martina Corrà, Marta Padovan, Giulia Cerretti, Mario Caccese, Antonio Silvani, Paola Gaviani, Diana Giannarelli, Gennaro Ciliberto, Marco G. Paggi

**Affiliations:** ^1^IRCCS - Regina Elena National Cancer Institute, Rome, Italy; ^2^Veneto Institute of Oncology IOV-IRCCS, Padua, Italy; ^3^IRCCS Besta Neurological Institute, Milan, Italy; ^4^Fondazione Policlinico Universitario A. Gemelli, IRCCS, Rome, Italy

**Keywords:** glioblastoma, drug repurposing, chlorpromazine, adjuvant treatment, *MGMT*

## Abstract

**Introduction:**

Drug repurposing is a promising strategy to develop new treatments for glioblastoma. In this phase II clinical trial, we evaluated the addition of chlorpromazine to temozolomide in the adjuvant phase of the standard first-line therapeutic protocol in patients with unmethylated *MGMT* gene promoter.

**Methods:**

This was a multicenter phase II single-arm clinical trial. The experimental procedure involved the combination of CPZ with standard treatment with TMZ in the adjuvant phase of the Stupp protocol in newly-diagnosed GBM patients carrying an unmethylated *MGMT* gene promoter. Progression-free survival was the primary endpoint. Secondary endpoints were overall survival and toxicity.

**Results:**

Forty-one patients were evaluated. Twenty patients (48.7%) completed 6 cycles of treatment with TMZ+CPZ. At 6 months, 27 patients (65.8%) were without progression, achieving the primary endpoint. Median PFS was 8.0 months (95% CI: 7.0-9.0). Median OS was 15.0 months (95% CI: 13.1-16.9). Adverse events led to reduction or interruption of CPZ dosage in 4 patients (9.7%).

**Discussion:**

The addition of CPZ to standard TMZ in the first-line treatment of GBM patients with unmethylated *MGMT* gene promoter was safe and led to a longer PFS than expected in this population of patients. These findings provide proof-of-concept for the potential of adding CPZ to standard TMZ treatment in GBM patients with unmethylated *MGMT* gene promoter.

**Clinical trial registration:**

https://clinicaltrials.gov/study/NCT04224441, identifier NCT04224441.

## 1 Introduction

Glioblastoma (GBM) is a frequent and severe brain tumor, characterized by poor response to treatment and an almost certainty of relapse. First-line GBM treatment, regardless of the molecular classification of the disease (1), consists in maximal surgical resection followed by radiotherapy with concomitant temozolomide (TMZ) treatment, followed by adjuvant TMZ. This scheme is however associated with a median overall survival (OS) of 14.6 months and a 5-year survival <5% (2), therefore leaving an unmet clinical need.

GBM presents high invasive and infiltrative properties (3), also coupled with a distinctive cellular heterogeneity, a prerequisite for a swift adaptation to treatment (4–7). Indeed, GBM has the ability to recover from genetic damages induced by radiotherapy and TMZ by means of an effective DNA repair system, especially in tumors characterized by unmethylated O6-methylguanine methyltransferase (*MGMT*) gene promoter (8). Of note, GBM is among the few tumors in which a single-drug treatment is currently in use: it may be possible to speculate that the addition of another therapy may help overcome resistance to TMZ (9).

An interesting characteristic of GBM is its responsiveness to neurotransmitters, as monoamines (10–12). The well-known interplay between neurons and tumors, especially GBM (11, 13), and the more recent identification of a synaptic neuron-GBM connectivity (14) confirms that neuron-secreted mediators are taken up by GBM cells, where they act as oncogenic stimuli (15). These findings paved the way for considering the addition of selected neuroleptic drugs as potential addition to GBM treatment (16). Indeed, many psychotropic drugs act on multiple postsynaptic receptors and display diverse pharmacological activity (16), including the ability to counteract GBM growth *in vitro* (17–19). Upon these assumptions, we evaluated the effects of one of the progenitors of neuroleptic medications, i.e., the antipsychotic drug chlorpromazine (CPZ), in use for about 70 years for psychiatric disorders in GBM patients. CPZ is a compound listed in the 2021 WHO Model List of Essential Medicines (20).

In an *in vitro* study, our group has already assayed CPZ effects on established and primary human GBM cells. The results defined the role of this drug in hindering GBM cell growth by acting at different levels through multimodal antitumor effects without any relevant action on non-cancer neuroepithelial cells (21, 22). In addition, CPZ acts synergistically with TMZ in hindering GBM vitality and stemness capabilities (21).

CPZ is a safe, low-cost and promptly available medication. All these conditions paved the way for repurposing CPZ as an add-on drug in GBM therapy. To this end, we planned the RACTAC (Repurposing the Antipsychotic drug Chlorpromazine as a Therapeutic Agent in the Combined treatment of newly diagnosed glioblastoma) phase II multicenter, single-arm study, in which CPZ is added to adjuvant TMZ in the first-line therapy of GBM patients whose tumor is characterized by an unmethylated *MGMT* gene promoter (8).

This study was designed with the purpose of investigating the clinical efficacy and safety of the addition of CPZ to the adjuvant TMZ administration in the first-line treatment of GBM patients carrying an unmethylated *MGMT* gene promoter.

## 2 Materials and methods

### 2.1 Study design

This was a multicenter, phase II single-arm clinical trial conducted in three Italian referral centers: the Regina Elena National Cancer Institute (Rome), the Veneto Institute of Oncology (Padua) and the Besta Neurological Institute (Milan).

**Figure 1:**
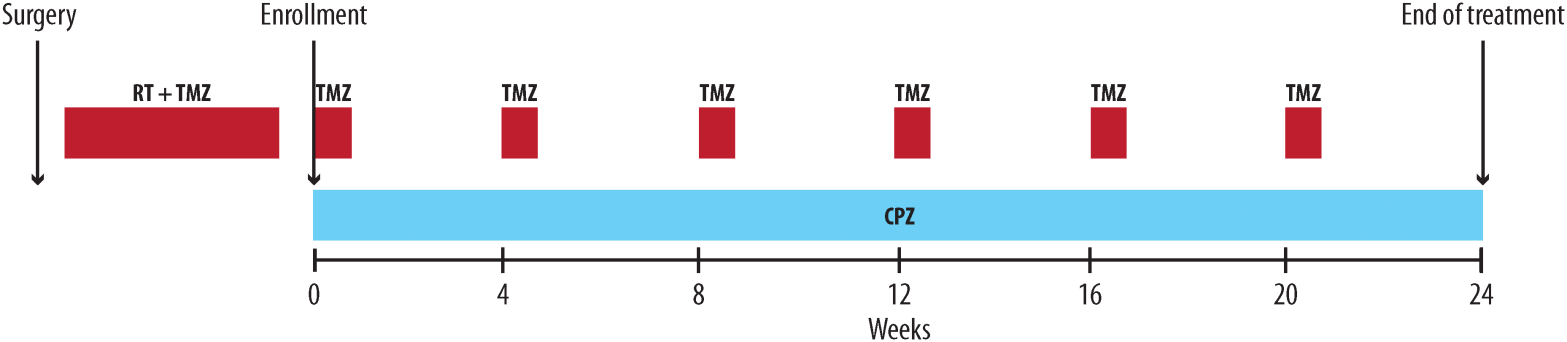
The RACTAC scheme: Scheme of the daily addition of CPZ during the adjuvant phase of the first line protocol for newly diagnosed GBM patients with unmethylated *MGMT* gene promoter. RT, radiotherapy; TMZ, temozolomide; CPZ, chlorpromazine.

The experimental procedure involved the combination of CPZ with standard treatment with TMZ in the adjuvant phase of the Stupp protocol (2) in newly diagnosed GBM patients carrying an unmethylated *MGMT* gene promoter. All patients received CPZ tablets [“Largactil”, Teofarma S.R.L., Valle Salimbene (PV), Italy], during TMZ adjuvant treatment at a starting dose of 25 mg/day orally from day 1 onwards. The dosage was increased to 50 mg/day at the second cycle of treatment, if well tolerated, and continued for 6 cycles or until disease progression, death, unacceptable toxicity, or consent withdrawal (**Figure 1**).

This clinical trial has been approved by our Institutional Ethics Committee on September 6, 2019, and is registered as EudraCT #2019-001988-75 and ClinicalTrials.gov Identifier #NCT0422444.

### 2.2 Eligibility

Patients aged 18-75 years with newly diagnosed and histologically confirmed supratentorial GBM (World Health Organization 2016) and unmethylated *MGMT* gene promoter status were eligible. *MGMT* gene promoter methylation status was assessed in a local laboratory for each participating center by pyrosequencing or methylation-specific PCR. Additional inclusion criteria were: gadolinium (Gd)-enhanced MRI 48 h after surgery; stable or decreasing dose of steroids for 1 week before enrolment; Karnofski performance status (KPS) ≥70; satisfactory laboratory blood and biochemical results within 2 weeks prior to enrolment.

### 2.3 Endpoints

Progression-Free Survival (PFS) was the primary endpoint of this study. PFS was defined as the time from the start of the Stupp regimen (2) to the earliest documented date of disease progression, based on Response Assessment in Neuro-Oncology (RANO) criteria (23, 24), as determined by the investigator, death due to any cause, or censored at the date of the last assessment. Evaluating a meta-analysis of 91 GBM clinical trials, the choice of PFS as an endpoint can be considered appropriate as a surrogate endpoint for earlier evaluation. The 6-month PFS appears correlated with 1-year overall survival (OS) and median OS (25).

The secondary endpoints of this study were: (i) Overall survival (OS); defined as the time from diagnosis to the date of death from any cause or loss-to-follow-up); (ii) combination treatment toxicity. Adverse events (AEs) were evaluated according to the National Cancer Institute’s Common Terminology Criteria for Adverse Events version 5.0. Safety assessments were performed from treatment initiation through 30 days after the last dose of chemotherapy with TMZ plus CPZ; (iii) Quality of Life (QoL), assessed by means of the EORTC QLQ C30+BN20 questionnaire at baseline and every 3 cycles (26, 27). Data on QoL are under evaluation at the time of drafting of this manuscript and will be presented in a separate paper.

### 2.4 Follow-up

Patients were followed-up monthly during adjuvant chemotherapy and every 3 months after completion of the standard treatment if the disease was stable. Radiological assessment was done by Gd brain MRI every 12 weeks from the first drug administration until progression.

Response assessment during study was based on investigators’ evaluation using the RANO criteria (23, 24), which included blinded interpretation of MRI scans, assessment of neurological status, KPS scores, and steroid use.

### 2.5 Statistical analysis

All analyses were performed for the patients who received at least one dose of the study treatment. The primary objective of the study was to evaluate the proportion of patients free from progression after 6 months (PFS-6). Considering as unacceptable a percentage of PFS-6 (P0) equal to 35% and a desirable PFS-6 of 55% (P1), a minimum of 41 patients would be needed to guarantee a power of 80% at a significance level of 5% (one-sided) (28). If at least 20 patients were progression-free after 6 months, the treatment would be considered sufficiently active. All survival curves were estimated using the Kaplan-Meier method.

Clinical and demographic characteristics are reported as absolute counts and percentage when related to categorical items and as median and range if referred to quantitative variables.

## 3 Results

### 3.1 Patient characteristics

Between April 2020 and August 2022, 45 patients were screened; 41 received at least one cycle of treatment and were included in the final analysis. Study follow-up was closed on December 31, 2022. Patients’ baseline characteristics and demographics are summarized in **Table 1**. The study was not adequately powered to determine whether there were any sex differences in clinical response to the treatment.

**Table 1:** Baseline characteristics of the enrolled GBM patients.

Variables	Study population (n= 41)
Age at diagnosis (Median, y)	56 (range 20-75)
Gender	M 29 (70.7%); F 12 (29.3%)
KPS median	90 (range 70-100)
Extent of resection %	Gross Total Resection (GTR) 41.5; Partial Resection (PR) 56.1; Biopsy 2.4
Steroid treatment at baseline (%)	21 (51%)
Dexamethasone dose (mean)	4 mg

KPS, Karnofsky Performance status; GTR, Gross Total resection.

Twenty patients (48.7%) completed 6 cycles of treatment with TMZ at standard dose plus CPZ; twenty-one patients (51.2%) discontinued the treatment due to early progression (median cycles completed = 5.5, range 2-6). The median follow-up was 15 months (range 3-37).

### 3.2 Progression-free survival

**Figure 2:**
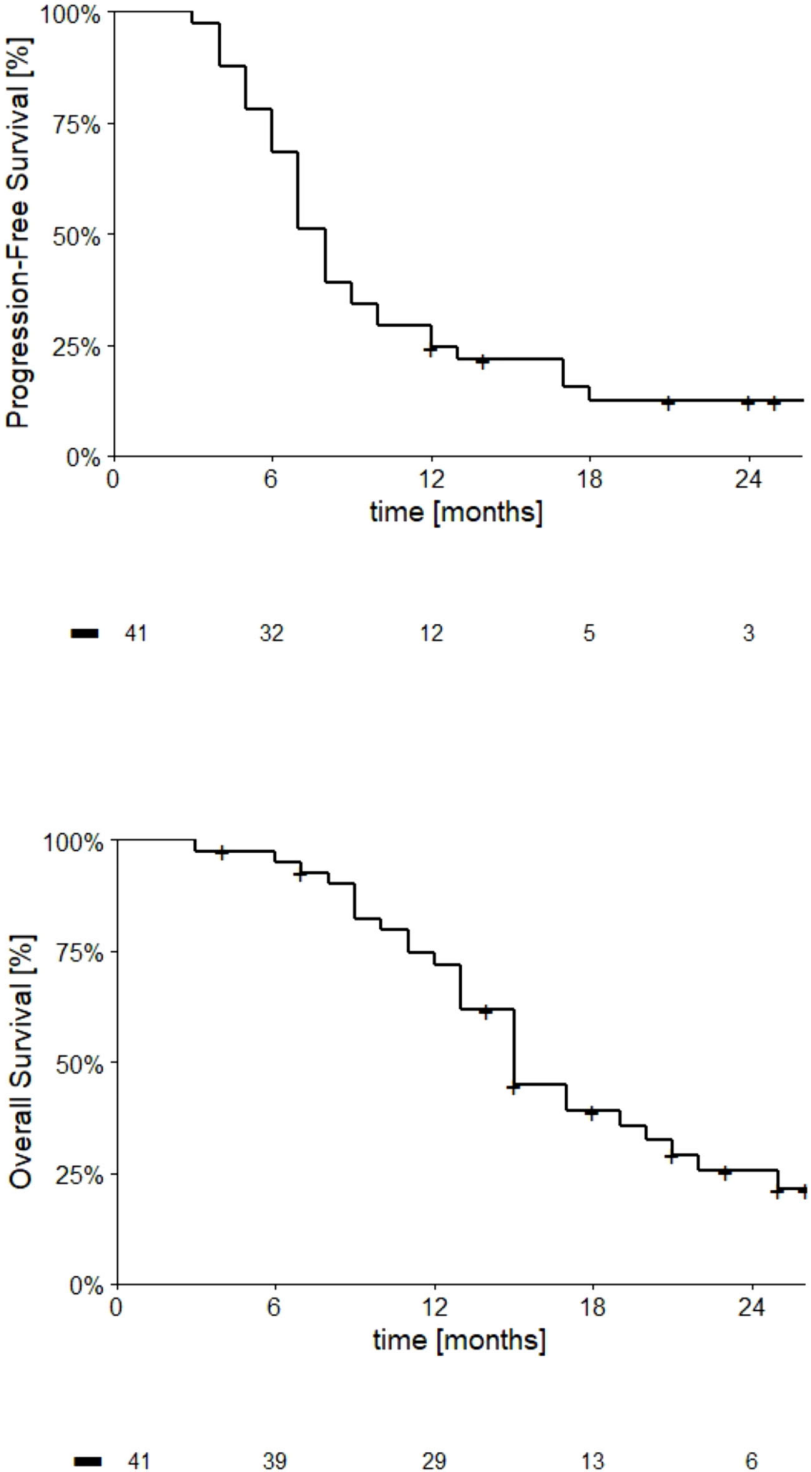
Kaplan-Meier curves describing PFS and OS trends (upper and lower charts, respectively) in GBM patients with unmethylated *MGMT* gene promoter treated according to the RACTAC protocol. The number of patients at risk at respective time points are indicated below the abscissa.

At 6 months, 27 (65.8%) patients were alive and without progression: thus, the primary endpoint was achieved. Overall, 35 patients (85.4%) experienced disease progression, with a median PFS of 8.0 months (95% CI: 7.0-9.0) (**Figure 2**). PFS at different time points is reported in **Table 2**.

**Table 2:** Progression-free survival (PFS) and overall survival (OS) in GBM patients with unmethylated *MGMT* gene promoter treated according to the RACTAC protocol.

	6 months	12 months	18 months	24 months
**PFS**	68.3%	24.4%	12.4%	12.4%
**OS**	95.1%	72.0%	38.9%	25.5%

### 3.3 Overall survival

At study closure, 29 deaths (69.7%) have been observed, and median overall survival was 15.0 months (95% CI: 13.1-16.9). OS at different time-points is reported in **Figure 2**; **Table 2**.

### 3.4 Role of the extent of resection

Patients who underwent a gross total resection (GTR) had a longer PFS and OS than patients who underwent a partial resection (PR). As described (29), the extent of resection is an important prognostic factor for survival in glioblastoma (GBM) patients. This is also true in our case series, which was developed on a chronological basis and is representative of the general population of these patients, with a prevalence of PRs plus one case of biopsy.

### 3.5 Toxicity

The treatment scheme was associated with the onset of an expected dose-dependent sedation, especially at the beginning of the CPZ administration, and liver toxicity (1 serious case), however expected for both CPZ and TMZ. Treatment was well tolerated in all patients with mild somnolence (grade 1-2) in 8 (19.5%) and asthenia in 12 (29.2%) patients. In three patients, grade 3-4 hyper-transaminasaemia was observed (7%). Adverse events led to reduction or interruption of CPZ dosage in 4 patients (9.7%). Decreased platelets count grade 3 was observed in one patient and neutropenia grade 3 in one. All these data are summarized in **Table 3**.

**Table 3:** Drug-related adverse events.

Adverse events	Grade 1-2	Grade >2
**Somnolence**	8	0
**Fatigue**	11	1
**Hypertransaminasemia**	1	2
**Thrombocytopenia**	6	1
**Neutropenia**	1	1

## 4 Discussion

GBM patients with unmethylated *MGMT* gene promoter comprise 55-60% of total GBM cases and have a poorer prognosis due to their intrinsic resistance to alkylating agents. At present, while it is well recognized that TMZ brings a benefit in patients with methylated *MGMT* gene promoter, its use in cases of unmethylated *MGMT* gene promoter is still controversial. Indeed, EANO evidence-based guidelines on diagnosis and treatment of diffuse gliomas of adulthood report that TMZ might only be effective in GBM patients with methylated *MGMT* gene promoter, whereas its effectiveness on patients with unmethylated *MGMT* gene promoter is modest (8, 30).

Various therapeutic strategies have been investigated in phase II clinical trials to overcome drug resistance or bypass DNA repair pathways in GBM patients with unmethylated *MGMT* gene promoter (8, 31). In the same setting, addition of drugs other than TMZ alone in the first-line treatment did not lead to encouraging results (32–34). Other studies evaluated the efficacy of new agents as replacements for TMZ in the first-line treatment of GBM patients with unmethylated *MGMT* gene promoter. For example, in the EORTC 26082 phase II trial, temsirolimus was administered either during radiotherapy or as adjuvant treatment, showing no clinical benefit (35). In a single arm phase II trial, enzastaurin, a protein kinase C inhibitor, administered before, concomitantly with, and after radiotherapy in GBM patients with unmethylated *MGMT* gene promoter, did not achieve the primary endpoint of PFS at 6 months (36). On the other hand, in the same population of GBM patients, bevacizumab plus irinotecan provided a better PFS at 6 months than TMZ, but with no improvement in OS (37).

Our results show that the addition of CPZ to standard TMZ in the first-line treatment of GBM patients with unmethylated *MGMT* gene promoter was safe and led to a longer PFS, i.e., 8.0, than expected in this patient population. This finding met the primary endpoint of the study. A recent meta-analysis of five phase III clinical trials found that the standard-of-care treatment for GBM patients with unmethylated *MGMT* gene promoter results in a PFS of 4.99 months and an OS of 14.11 months (38).

In our study, however, median OS was 15 months, thus not representing a clinically relevant improvement over the data reported in this meta-analysis. Such an outcome could also relate to the administration of CPZ only in the adjuvant phase of treatment. Further studies are needed to investigate the concomitant association of CPZ also during radiotherapy.

The safety profile of CPZ was consistent with its well-known pharmacological profile, even when administered concomitantly with TMZ. In our study, the most frequent adverse event were mild somnolence (grade 1-2) in 18% of patients, usually in the first month of treatment, and fatigue, observed in 30% of patients, a symptom commonly reported within a range of 40-70% in primary brain tumor patients (39).

Quality of life (QoL) is an important outcome to be evaluated in these GBM patients. In our study, it has been measured by means of the EORTC QLQ-C30 and QLQ-BN20 questionnaires. These evaluations are currently in progress, and the results will be published shortly.

Most relevant limitations of the RACTAC trial are the small number of patients included in the study and the lack of a control group. However, the clinical characteristics of the patients at baseline appear representative of the real-world population of unmethylated *MGMT* gene promoter GBM. The PFS observed in this trial was 8.0 months and resulted promisingly longer than that reported in previous published trials in this population.

CPZ is a well-known DRD2 antagonist (40) and therefore has been successfully used in the treatment of psychiatric disorders. We intended to take advantage of its ability to interfere with the function of DRD2, as well as a number of other neuromediator receptors (https://go.drugbank.com/drugs/DB00477), to hamper the pseudo-synaptic, oncogenic interplay between neurons and GBM. In addition, it should be considered that there is also a profound interplay between peripheral/central nervous systems and cancer, which acts through monoamine neuromediators and could represent a vulnerability targetable through the use of repurposed neuropsychiatric drugs in oncology and appliable to diverse cancer types (41, 42).

Evaluating the features of CPZ possibly useful in GBM treatment, we could also consider a number of studies showing the ability of this drug to hinder GBM malignant features in preclinical settings (43). In the same context, during the course of the RACTAC clinical trial, our group further refined the pharmacological effects of CPZ on GBM cells *in vitro*, demonstrating the ability of this medication to hinder GBM malignant features at multiple levels (21, 22) other than its known interference with the activity of neurotransmitters.

Since the plasticity of GBM and its ability to remodulate its cell population based on the selective pressure generated by therapies, we can state that this tumor cannot be defined as a “single-path disease”, being therefore quite unsatisfying to treat it by means of targeted therapies. On these premises, it appears reasonable to consider the opportunity to use “dirty drugs”, i.e., drugs that are not too targeted, but are able to hit some generalized vulnerabilities characterizing cancer cells.

Considering the escalation of the costs of novel anticancer medications, the long time it takes for them to reach the market and the consequent nonavailability for a great number of patients, the use of repurposed drugs can dramatically cut down time and drug expenses for effective medications to reach the bedside, with significant benefits for the patients and the Health Systems. In addition, the characteristics inherent in repositionable drugs represent a further therapeutic chance for GBM patients for which no second-line therapy is currently established as effective or for those that have already experienced all known therapeutic opportunities.

The RACTAC phase II clinical study was designed to investigate whether adding CPZ to the standard adjuvant TMZ in the Stupp protocol could improve therapeutic efficacy in patients with GBM with an unmethylated *MGMT* gene promoter. It also assessed the tolerability of adding a neuroleptic medication to GBM patients after neurosurgery and combined chemo-radiotherapy. This clinical trial is a proof-of-concept for the effectiveness of interfering with oncogenic monoamine signaling between neurons and GBM to inhibit tumor growth and malignancy, and, although not exhaustive, it can support the initiation of a subsequent phase III randomized clinical study.

## Data availability statement

The datasets presented in this study can be found in online repositories. The names of the repository/repositories and accession number(s) can be found below: GARR repository at the following link: https://gbox.garr.it/garrbox/s/HZ2g4aBD26lJh1e.

## Ethics statement

The studies involving humans were approved by Comitato Etico Centrale IRCCS – Sezione IFO-Fondazione Bietti, Roma (EudraCT # 2019-001988-75; ClinicalTrials.gov Identifier: NCT04224441). The studies were conducted in accordance with the local legislation and institutional requirements. The participants provided their written informed consent to participate in this study. Written informed consent was obtained from the individual(s) for the publication of any potentially identifiable images or data included in this article.

## Author contributions

AP: Conceptualization, Data curation, Methodology, Resources, Supervision, Validation, Writing – original draft, Writing – review & editing. GL: Data curation, Resources, Validation, Writing – review & editing. VV: Data curation, Writing – review & editing. DB: Data curation, Writing – review & editing. CA: Data curation, Writing – review & editing. IC: Data curation, Resources, Writing – review & editing. MCo: Data curation, Resources, Writing – review & editing. MP: Data curation, Resources, Writing – review & editing. GCe: Data curation, Resources, Writing – review & editing. MCa: Data curation, Resources, Writing – review & editing. AS: Data curation, Writing – review & editing. PG: Data curation, Resources, Writing – review & editing. DG: Data curation, Formal analysis, Writing – review & editing. GCi: Funding acquisition, Supervision, Writing – review & editing. MGP: Conceptualization, Data curation, Funding acquisition, Methodology, Resources, Supervision, Validation, Writing – original draft, Writing – review & editing.
